# Analysis of Cultivar-Specific Variability in Size-Related Leaf Traits and Modeling of Single Leaf Area in Three Medicinal and Aromatic Plants: *Ocimum basilicum* L., *Mentha* Spp., and *Salvia* Spp.

**DOI:** 10.3390/plants9010013

**Published:** 2019-12-20

**Authors:** Maurizio Teobaldelli, Boris Basile, Francesco Giuffrida, Daniela Romano, Stefania Toscano, Cherubino Leonardi, Carlos Mario Rivera, Giuseppe Colla, Youssef Rouphael

**Affiliations:** 1 Department of Agricultural Sciences, University of Naples Federico II, 80055 Portici, Italy; maurizio.teobaldelli2@unina.it (M.T.);; 2 Department of Agriculture, Food and Environment, University of Catania, 95100 Catania, Italy; francesco.giuffrida@unict.it (F.G.); dromano@unict.it (D.R.); stefania.toscano@unict.it (S.T.); cherubino.leonardi@unict.it (C.L.); 3 Department of Agriculture and Forest Sciences, Tuscia University, 01100 Viterbo, Italy; carlos.biogas@gmail.com

**Keywords:** basil, calibration, cultivars, leaf phenotypic traits, mint, plant modeling, principal component analysis, sage, validation

## Abstract

In this study, five allometric models were used to estimate the single leaf area of three well-known medicinal and aromatic plants (MAPs) species, namely basil (*Ocimum basilicum* L.), mint (*Mentha* spp.), and sage (*Salvia* spp.). MAPs world production is expected to rise up to 5 trillion US$ by 2050 and, therefore, there is a high interest in developing research related to this horticultural sector. Calibration of the models was obtained separately for three selected species by analyzing (a) the cultivar variability—i.e., 5 cultivars of basil (1094 leaves), 4 of mint (901 leaves), and 5 of sage (1103 leaves)—in the main two traits related to leaf size (leaf length, L, and leaf width, W) and (b) the relationship between these traits and single leaf area (LA). Validation of the chosen models was obtained for each species using an independent dataset, i.e., 487, 441, and 418 leaves, respectively, for basil (cv. ‘Lettuce Leaf’), mint (cv. ‘Comune’), and sage (cv. ‘Comune’). Model calibration based on fast-track methodologies, such as those using one measured parameter (one-regressor models: L, W, L^2^, and W^2^) or on more accurate two-regressors models (L × W), allowed to achieve different levels of accuracy. This approach highlighted the importance of considering intra-specific variability before applying any models to a certain cultivar to predict single LA. Eventually, during the validation phase, although modeling of single LA based on W^2^ showed a good fitting (R^2^_basil_ = 0.948; R^2^_mint_ = 0.963; R^2^_sage_ = 0.925), the distribution of the residuals was always unsatisfactory. On the other hand, two-regressor models (based on the product L × W) provided the best fitting and accuracy for basil (R^2^ = 0.992; RMSE = 0.327 cm^2^), mint (R^2^ = 0.998; RMSE = 0.222 cm^2^), and sage (R^2^ = 0.998; RMSE = 0.426 cm^2^).

## 1. Introduction

In modern horticulture, growers need to optimize plant development and yield [1] in order to meet the food demand of increasing populations [2], especially in developing countries [3], and contribute towards food security and social stability. Within this scope, applied research on innovative horticultural practices can make effective use of dynamic crop growth models [4] under conditions optimal for plant growth and for eliciting plant response to abiotic stresses [5], therefore, allowing a more rational use of resources, such as water and nutrients [4].

Several fundamental physiological processes such as photosynthesis, transpiration, and cooling are facilitated by leaves [6] and they are, therefore, strongly influenced by leaf morphology (size, shape, symmetry, venation, organization, and petiole characteristics) [7]. The characterization of leaf morphology and quantification of leaf area (LA) and/or leaf area index (LAI) is consequently of paramount importance to horticultural crop science. In this respect, there is an increasing interest in using computer-assisted imaging systems [8] for producing reliable biometric measurements [9] and analyzing phenotypic traits related to plant architecture and leaf characteristics [10]. For instance, data on leaf characteristics can be incorporated into databases [11,12] and employed to validate time-series quantification of leaf morphology (e.g., [13,14]) and to determine the performance of computer-assisted imaging systems and machine learning algorithms used to classify/recognize phenotypic traits of specific genotypes [15]. 

Leaf area is generally measured with destructive or non-destructive methods [16], the latter often preferred as they are faster, cheaper, and non-invasive (i.e., no excision of leaves is required), therefore, permitting repeated and simultaneous measurements of LA and other physiological parameters (e.g., leaf gas exchange or fluorescence) on the same leaves.

Collected information, such as leaf blade length (L) and width (W) [17,18,19,20,21,22,23,24,25] or the shape ratio of the leaf (L:W) [26], can be useful for characterizing leaf functions and structure, based only on proxy variables. In particular, the leaf shape ratio is of particular importance in horticultural sciences as it is regulated by several genetic factors and mutations [27], whose diversity can be analyzed in functional [28] and evolutionary terms [29]. 

Thus far, numerous models have been proposed and applied with respect to both leaf (e.g., [20,30,31]) and shoot level [31,32,33,34,35,36,37,38,39,40,41] morphology of several fruit, vegetable, ornamental, medicinal, and aromatic crops [42]. Currently, LA models for aromatic and medicinal plants comprise several species such as basil, winter red Bergenia, or purple bergenia, calamint, coffee, cherry laurel, bush-willows, jimson weed, wild cucumber, horse-eye bean, lemon balm, peppermint, oleander, mountain mint, opium poppy, ground-cherry, or winter cherry, picrorhiza or kutka, saffron, sugar leaf, snowbell, summer snowflake, tea, common nettle, orange mullein [42], valeriana [43], and pepper plants [44]. 

The world production of medicinal and aromatic plants (MAPs) is expected to rise up to 5 trillion US$ by 2050 [45]. Thanks to their aromatic oils [45] and other phytochemical constituents MAPs are used (a) to deter herbivores, pathogens, and parasites, (b) as culinary herbs and spices (e.g., thyme, laurel, and basil), (c) to produce scent and (d) as ornamentals (e.g., *Eucalyptus* spp., *Lavandula* spp., and *Cistus* spp.). In this context, there is an increasing interest in collecting systematic information on the physiology and phenotypic traits of these crops, information that can be used, for instance, to simulate seasonal variations of leaf area and, therefore, estimate periodic treatments and the needs for irrigation during the cultivation of MAPs. In addition, precise estimations of LA will be necessary to develop more complex process-based plant growth models that can be used to build support decision systems to help growers in managing fertilization and irrigation. Therefore, the aims of this work were to employ rigorous statistical analysis in order to (1) test and compare fast but accurate generalized allometric models for different cultivars of basil, mint, and sage—three well-common MAPs used worldwide as culinary herbs—and (2) validate the best models using an independent dataset.

## 2. Results

### 2.1. Phenotypic Traits

Data analysis for the three aromatic species facilitated the characterization of variability in the main leaf phenotypic traits (length, width, and leaf shape ratio) observed on different cultivars (Table 1). Regarding basil, the cultivar ‘Mammoth’ showed the highest maximum values of both leaf L (7.66 cm) and leaf W (7.05 cm), whereas the cultivars ‘Lettuce Leaf’ (used as independent dataset during the validation of the chosen model) and ‘Cinnamon’ had the lowest minimum values of the leaf length (1.65 cm) and leaf width (1.06 cm), respectively. The highest maximum leaf shape ratio was measured in the cultivar ‘Purple Petra’ (2.41), whereas the lowest minimum shape ratio (1.03) was recorded in the cultivar ‘Lettuce Leaf’ (Table 1).

Within the mint species, the cultivar ‘Comune’ showed the maximum leaf L (7.47 cm), whereas the cultivar ‘Glaciale’ had the highest maximum value of leaf W. The cultivar ‘Glaciale’ also had the lowest minimum leaf length (1.32 cm), whereas the cultivar ‘Suaveolens’ showed the lowest minimum leaf width (0.78 cm). The cultivar ‘Comune’ also showed the lowest minimum leaf shape ratio value (1.00), whereas the cultivar ‘Piperita’ (2.42) had the highest maximum L:W value (Table 1). 

For the sage, the longest and largest leaves were found in the cultivar ‘Maxima’ (max L = 12.61 cm; max W = 7.11 cm) whereas the cultivars ‘Comune’ and ‘Fariancea’ showed the lowest minimum values for L (1.52 cm) and W (0.58 cm), respectively. Finally, the highest maximum leaf shape ratio (4.16) was measured in the cultivar ‘Fariancea’, whereas the cultivar ‘Tricolor’ (1.22) had the lowest minimum L:W ratio (Table 1). 

### 2.2. Principal Component Analysis

Very high correlations (r > 0.95; Table 2) between LA and W were observed in 13 cultivars out of the 14 analyzed (calibration experiment), whereas similar correlations were found between LA and L in 11 cultivars. However, it should be noted that in both basil and sage, LA was better correlated to W rather than to L (Table 2). On the contrary, in mint, better correlations were found between LA and L rather than between LA and W (3 cultivars out of the 4 analyzed; Table 2).

**Figure 1:**
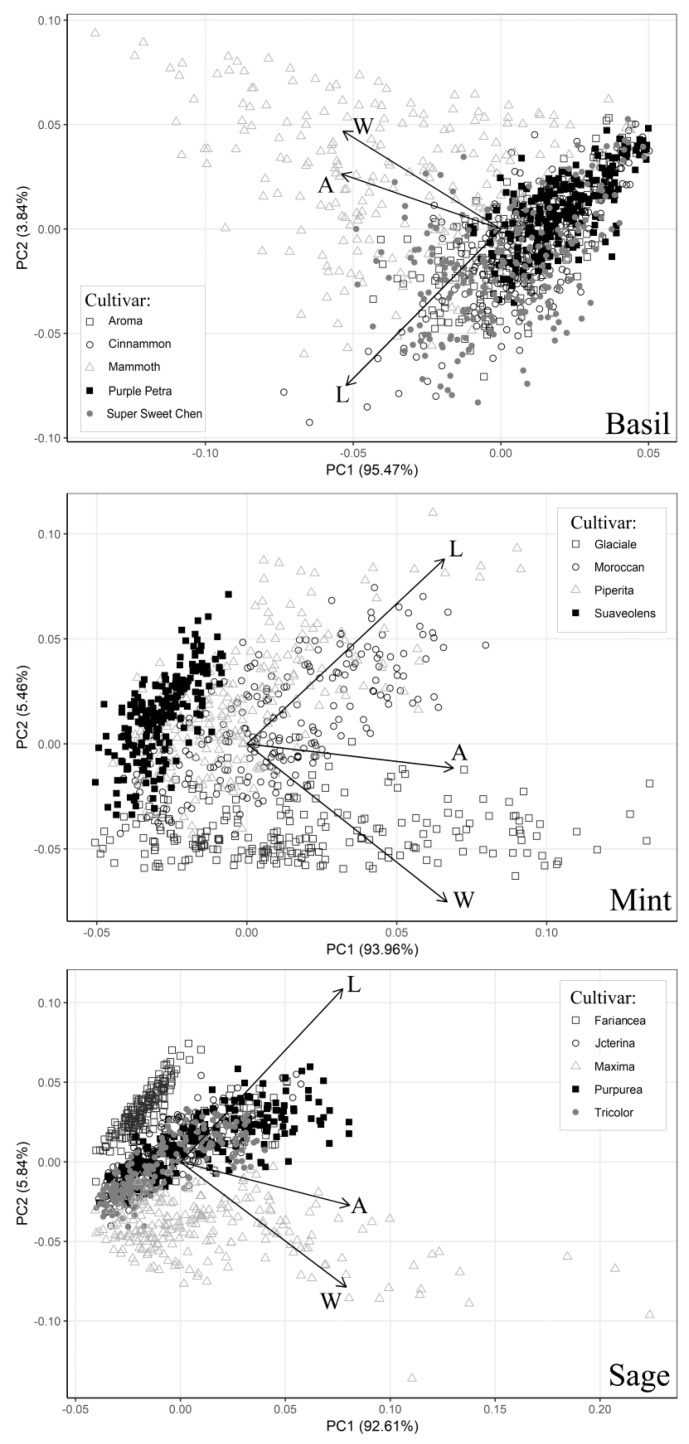
Main outputs from the principal component analysis carried out using three aromatic species (basil, mint, and sage) and 14 different cultivars (calibration experiment). The bipolar plot was build using two main factors (PC1 and PC2, see Table 3 for more details). Loadings, representing the main dependent (LA) and independent variables (L, W) used in this study, were also reported. LA = single leaf area (cm^2^); L = leaf length (cm); W = leaf width (cm).

The first two principal components (PC1 and PC2) of the PCA, carried out using leaves collected on the 14 cultivars used for calibration, explained 99.3%, 98.5%, and 98.4% of the total variance for basil, mint and sage, respectively (Table 3). For basil, PC1 had negative associations (loadings) with the three variables, whereas for mint and sage, PC1 had positive loadings with LA, L and W. Similarly, the associations between PC2 and the three variables were quite similar in mint and sage, whereas the loadings always had an opposite sign in basil (Table 3). As a result, the vectors obtained with the PCA were very similar in mint and sage, while in basil, they had the opposite direction (Figure 1). In addition, in the three PCA biplots, the vectors LA and W were spatially closer to each other compared to the vectors LA and L. This was particularly true for basil. Eventually, it is interesting to note how in some cultivars (i.e., ‘Mammoth’, ‘Glaciale’ and ‘Maxima’) the maximum variability was mainly along the axes of loading ‘W’ whereas in other cultivars (Cinnamon’, ‘Moroccan’ and ‘Piperita’) this occurred along the axes of the ‘L’ loading (Table 3).

Considering the eigenvalue of each factor (Table 3), only PC1 contributed significantly to the overall variances, whereas PC2 and PC3 contributed little to the explanation of variances in the three variables and, therefore, may be considered redundant and be ignored.

### 2.3. Model Calibration

**Figure 2:**
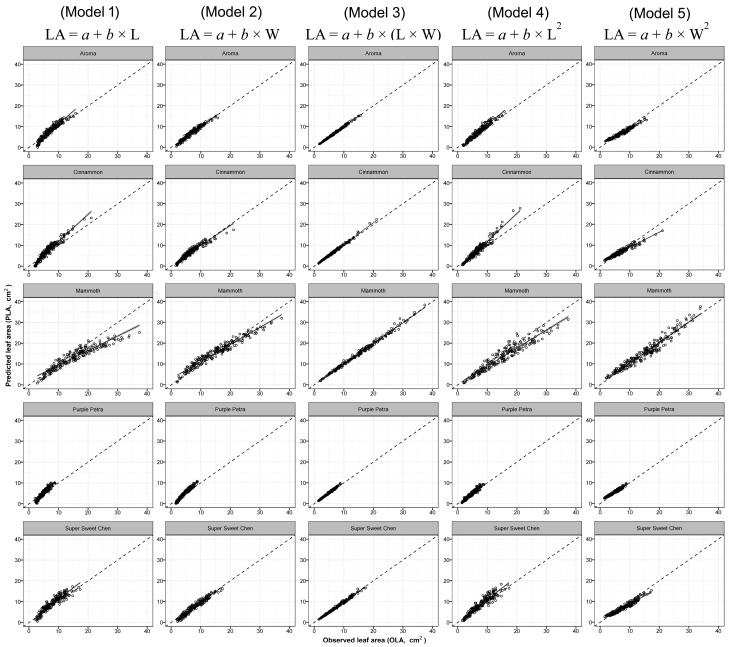
Predicted leaf area (PLA) using model 1 to 5 (see Table 6) obtained with pooled data of 5 different basil cultivars, vs. observed values of single leaf area (OLA) of each cultivar used in the calibration experiment. Dotted lines represent the 1:1 relationship between the predicted and observed values. The solid line and grey area represent, respectively, linear regression line of each model and the smoothing function based on generalized linear model (GLM).

**Figure 3:**
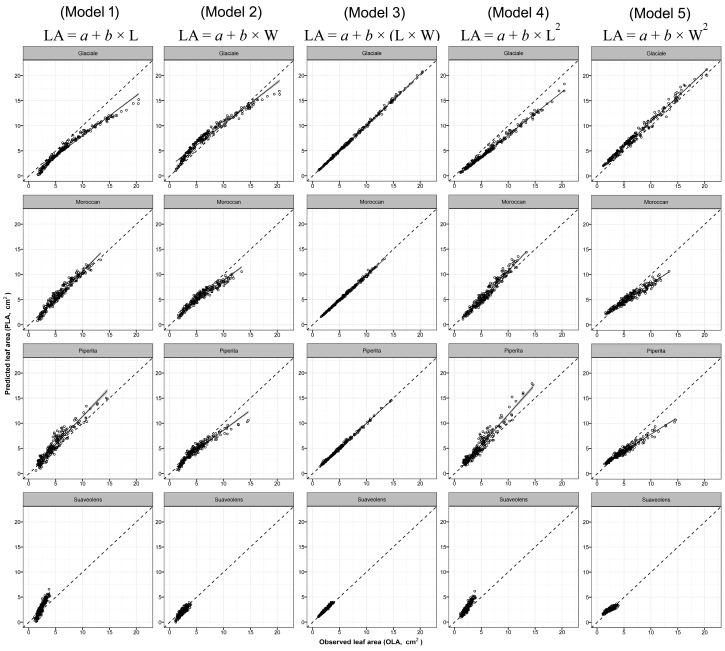
Predicted leaf area (PLA) using model 1 to 5 (see Table 6) obtained with pooled data of 4 different mint cultivars vs. observed values of single leaf area (OLA) of each cultivar used in the calibration experiment. Dotted lines represent the 1:1 relationship between the predicted and observed values. The solid line and grey area represent, respectively, linear regression line of each model and the smoothing function based on generalised linear model (GLM).

**Figure 4:**
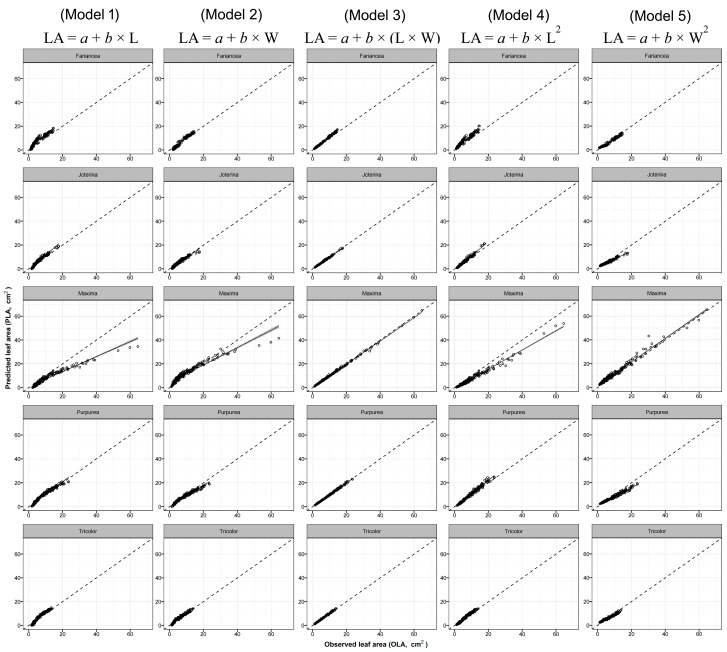
Predicted leaf area (PLA) using model 1 to 5 (see Table 6) obtained with pooled data of 5 different sage cultivars, vs. observed values of single leaf area (OLA) of each cultivar used in the calibration experiment. Dotted lines represent the 1:1 relationship between the predicted and observed values. The solid line and grey area represent, respectively, linear regression line of each model and the smoothing function based on generalised linear model (GLM).

Overall, all models predicted LA values of the three selected aromatic species with high accuracy (R^2^_basil_ = 0.850–0.995; RMSE_basil_ = 0.40–4.43 cm^2^; R^2^_mint_ = 0.861–0.997; RMSE_mint_ = 0.17 cm^2^; R^2^_sage_ = 0.791–0.994; RMSE_sage_ = 0.45–2.69 cm^2^). All the predicted intercepts and regressors were statistically significant, except the intercept of model no. 3 for sage species that was not significant (Table 4). Differences in the fitting capability and accuracy were found in the five models depending on whether the prediction was based on one-regressor (L, W, L^2^, or W^2^) or two-regressors (L × W) algorithms. Although the calibration of the one-regressor model carried out with pooled data permitted to obtain high R^2^ and low RMSE values (Figure 2, Figure 3 and Figure 4), it should be outlined that, in some cases and with certain cultivars, one-regressor models were unable to effectively predict the single LA values of some of the cultivars used in the calibration phase (model parameterization). Nevertheless, between the four one-regressor models, the best fittings (R^2^_basil_ = 0.956; R^2^_mint_ = 0.925; R^2^_sage_= 0.937), accuracy (RMSE_basil_ = 1.14 cm^2^; RMSE_mint_ = 0.89 cm^2^; RMSE_sage_ = 1.48 cm^2^) and ranking (BIC_basil_ = 3413.3; BIC_mint_ = 2374.6; BIC_sage_ = 4011.7) were obtained using model no. 5 based on squared leaf width (W^2^) values (Table 4).

Conversely, using the model no. 3 based on two proxy parameters (L × W), the prediction of the single LA of each cultivar used in the calibration was always accurate (Table 4, Figure 2, Figure 3 and Figure 4) (R^2^_basil_ = 0.995; RMSE_basil_ = 0.40 cm^2^; R^2^_mint_ = 0.997; RMSE_mint_ = 0.17 cm^2^; R^2^_sage_ = 0.994; RMSE_sage_ = 0.45 cm^2^), resulting 1st ranking for the BIC (BIC_basil_ = 1122.6; BIC_mint_ = −620.9; BIC_sage_ = 1373.2) criterion.

Finally, coefficients of the first ranking models (models no. 3 and no. 5) obtained during the calibration phase, were refitted using non-parametric bootstrap analysis (Table 5). The bias of the bootstrapped coefficients (intercept and regressors) computed using model no. 3 were on average 0.05%, 0.49%, and 2.29% of the original values respectively for basil, mint, and sage. Instead, by using model no. 5 the bias of the bootstrapped coefficients (intercept and regressors) were on average 0.33%, 0.14%, and 0.18% of the original values respectively for basil, mint, and sage (Table 5).

### 2.4. Model Validation

**Figure 5:**
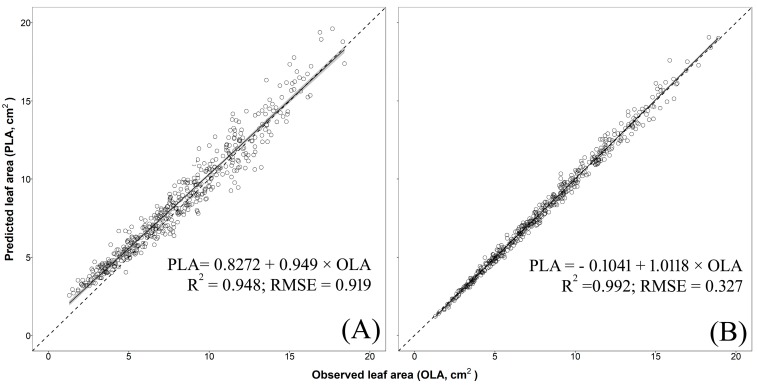
Predicted leaf area (PLA) estimated using (**A**) one-regressor bootstrapped model no. 5 [LA = 1.603 + 0.803 × W^2^] and (**B**) two-regressors bootstrapped model no. 3 [LA = −0.125 + 0.713 × (L × W)] vs. observed values of single leaf areas (OLA) of basil cv. ‘Lettuce Leaf’ (validation experiment). The solid line and the grey area represent, respectively, linear regression line of bootstrapped models no. 3 and no. 5 and the smoothing function based on the generalized linear model (GLM). R^2^ and residual standard error (RMSE in cm^2^) are also reported. Dotted lines represent the 1:1 relationship between the predicted and observed values.

**Figure 6:**
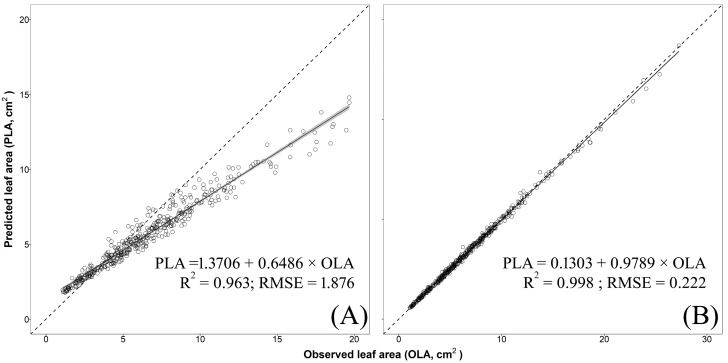
Predicted leaf area (PLA) estimated using (**A**) one-regressor bootstrapped model no. 5 [LA = 1.058 + 0.782 × W^2^] and (**B**) two-regressors bootstrapped model no. 3 [LA = 0.030 + 0.739 × (L × W)] vs. observed values of single leaf areas (OLA) of mint cv. ‘Comune’ (validation experiment). The solid line and the grey area represent, respectively, linear regression line of bootstrapped models no. 3 and no. 5 and the smoothing function based on the generalized linear model (GLM). R^2^ and residual standard error (RMSE in cm^2^) are also reported. Dotted lines represent the 1:1 relationship between the predicted and observed values.

**Figure 7:**
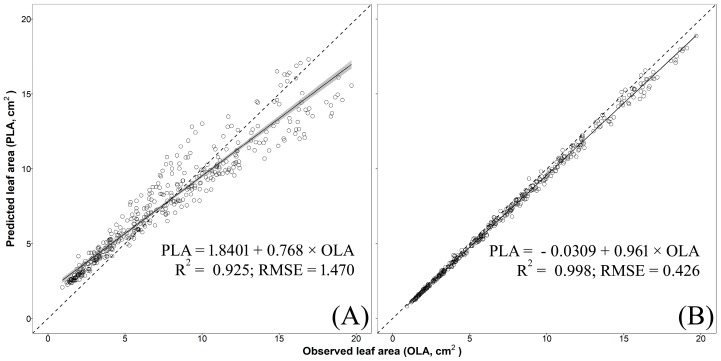
Predicted leaf area (PLA) estimated using (**A**) one-regressor bootstrapped model no. 5 [LA = 1.308 + 1.269 × W2] and (**B**) two-regressors bootstrapped model no. 3 [LA = −0.035 + 0.723 × (L × W)] vs. observed values of single leaf areas (OLA) of sage cv. ‘Comune’ (validation experiment). The solid line and the grey area represent, respectively, linear regression line of bootstrapped models no. 3 and no. 5 and the smoothing function based on a generalized linear model (GLM). R^2^ and residual standard error (RMSE in cm^2^) are also reported. Dotted lines represent the 1:1 relationship between the predicted and observed values.

The validation of models no. 3 (two-regressors) and no. 5 (one-regressor) were carried out, for each species, using an additional independent cultivar. The analysis showed that model no. 3 was able to effectively and accurately predict single LA of basil cv. ‘Lettuce Leaf’ (R^2^ = 0.992, RMSE = 0.327 cm^2^, Figure 5B) and mint cv. ‘Comune’ (R^2^ = 0.998, RMSE = 0.222 cm^2^, Figure 6B). A satisfactory residual dispersion pattern was found in both cases with almost all the points scattered randomly around the zero residual horizontal line and comprised between the upper and lower limits of agreement and with no visible pattern to the points (Figures S1B and S2B). Conversely, model no. 3 underestimated, especially for big leaves, single LA of sage cv. ‘Comune’ (R^2^ = 0.998; RMSE = 0.426 cm^2^; Figure 7B) with points of the residual plot positioned mostly above the graph of the prediction equation (Figure S3B).

Model no. 5 showed a moderate capability to predict single LA of basil (R^2^ = 0.948; RMSE = 0.919 cm^2^; Figure 5A) with an overestimation for small-to-medium leaves (LA < 8 cm^2^) and this led to an unsatisfactory distribution of the residuals (Figure S1A). When this model was used for mint and sage, a greater dispersion of the predicted values around the 1:1 line and highest RMSE were found (mint: R^2^ = 0.963, RMSE = 1.876 cm^2^, Figure 6A; mint: R^2^ = 0.925, RMSE = 1.470 cm^2^, Figure 7A), with an overestimation for the small leaves (especially in sage; Figure 7A) and a large underestimation for medium-to-large leaves in both species (Figure 6A and Figure 7A). This led to a fully unsatisfactory distribution of the residuals (Figures S2A and S3A).

## 3. Discussion

The importance of this study lies in the fact that the leaf and, by extrapolation, the entire canopy represent the fundamental physiological hubs of photosynthesis and of gas exchange with the atmosphere. As the ability to intercept light is clearly dependent on the two-dimensional leaf structure (i.e., shape and area) [27], the characterization of leaf L and W in several species has a large value within the broad fields of botany, plant physiology, and crop science. This information can be also needed in the future to determine the performance of novel photogrammetry and computer vision algorithms used to characterize whole-plant phenotypic traits of specific genotypes [15]. In this study, species-specific data on phenotypic traits (leaf L and W) of basil, mint, and sage were collected and, following rigorous statistical analysis, they were used to calibrate and validate five single leaf area (LA) allometric models. 

To be effective, a single leaf model needs to be accurate over a large range of variability of phenotypic traits, a condition that is attainable by (a) using data for calibration collected on a large number of cultivars, and (b) validating the final model using additional independent dataset. For this reason, we gave particular importance to the preliminary characterization of phenotypic traits by using three different species and 14 cultivars (5, 4, and 5 cultivars for basil, mint, and sage, respectively), and by choosing three additional cultivars to validate the best ranking models. As reported by Tsukaya [28], leaf W and narrow leaf shape (larger leaf index = stenophylly) might be driven evolutionarily by the growth habitat and other external conditions experienced by a species. In this context, many factors and different genes are thought to be involved in the selection of a particular leaf W for any given species and, therefore, as a final result, in the evolution of the leaf shape index [28]. All the species analyzed in this study belong to the *Lamiaceae*, a family having as the main center of variability the Mediterranean basin generally in degraded areas such as maquis and garrigues with rocky, calcareous, or sandy soils. In this case, the leaf shape ratio estimated on pooled data (calibration sets; Table 6) showed that sage had a narrow leaf (L:W = 2.4) and the largest variability, whereas basil and mint cultivars showed a quite similar leaf shape ratio with a mean value of about 1.5. At the same time, the PCA highlighted the existence, in some cultivars, of a good correlation between leaf W and single LA, thus explaining the better fitting and higher accuracy showed in some cases by the quadratic model based on the squared leaf W parameter (model no. 5).

The PCA also highlighted, for some cultivars, a good correlation (especially in mint) between the single LA and leaf L. These results were consistent with what observed graphically using PC1 and PC2. Indeed, some cultivars were distributed principally along with the leaf L loading, whereas others were either associated with the leaf W loading or mainly concentrated near the origin of the axes (Figure 1).

Comparing the PCA graphs with the ability of the five models to predict the single LA of the calibration set cultivars, it is clear that the observed intra-specific variability reduced the effectiveness of the one-regressor models. Moreover, during the validation phase, the one-regressor model no. 5 performed moderately well only for basil, but when it was used for mint and sage, blade size of small and large leaves was overestimated and underestimated, respectively. Indeed, the residual plots (Figures S2A and S3A) confirmed that model no. 5 was not a good fit, at least for mint and sage species. Although the results showed the limitation of models based on a fast single measurement (in particular in mint and sage) at the expense of a slightly increased variation, one-regressor models based on leaf W can be used in calibrated cultivars, especially if a range of different growing conditions (e.g., open-field and greenhouse) and situations are considered and included during the calibration and validation phases. This is also consistent with previous results reported by Gao et al. [9] for rose genotypes and by Teobaldelli et al. [46] for Loquat. On the contrary, a single leaf area could be estimated using the product of L and W without calibration per genotype, as reported by Rouphael et al. [30] and Teobaldelli et al. [46]. All these important aspects were also confirmed in our study for the three analyzed MAPs species. Indeed, our results outlined the importance of the two-regressors model (no. 3) based on the product of L × W, that was able to provide accurate predictions of the single LA both for all the three species and for each cultivar during the calibration and validation phases. This result is consistent also with previous studies on LA estimation in several fruits and horticultural crops ([18,36,47,48,49]). 

Our study was carried out using healthy leaves collected on unstressed plants grown in a greenhouse. Thus, it cannot be excluded that the models developed in our study will need to also be validated under other growing conditions and under different intensity levels of biotic and abiotic stresses. However, previous studies [50] carried out on eggplants reported that models calibrated using cultivars grown under open-field conditions might provide also good results if validated against data collected in greenhouses.

## 4. Materials and Methods 

### 4.1. Growth Conditions, Plant Material, and Data Collection

A greenhouse experiment was carried out during the 2011 spring at the private farm Gli Aromi (36°45′42.50″ N 14°42′36.89″ E) located in Sicily, southern Italy. The tested crops for the present study were 3 medicinal and aromatic plants (MAPs) species, namely basil (*Ocimum basilicum* L.), mint (*Mentha* spp.), and sage (*Salvia* spp.). Plants were grown according to the same commercial protocol and under identical natural light conditions. Inside the greenhouse, the mean air temperature was 24 °C, varying between 19 and 31 °C and the relative humidity was 60% and 75% during day and night, respectively. The tested medicinal and aromatic plants were grown in plastic containers (diameter: 14 cm; height: 12 cm) containing a peat/perlite mixture in a 1:1 volume ratio. 

For model calibration, the trial included a total of 5 basil cultivars (‘Aroma’, ‘Cinnamon’, ‘Mammoth’, ‘Purple Petra’ and ‘Super Sweet Chen’; four mint cultivars (‘Moroccan’ [*Mentha spicata* L.], ‘Piperita’ [*Mentha × piperita* L.], ‘Glaciale’ [*Mentha × rotundifolia* (L.) Huds. and ‘Suaveolens’ [*Mentha suaveolens* Ehrh.]); and five sage cultivars (‘Jcterina’, ‘Tricolor’ and ‘Maxima’ [*Salvia officinalis* L.], ‘Purpurea’ [*Salvia purpurea* Cav.] and ‘Fariancea’ [*Salvia farinacea* Benth.]). For model validation, the ‘Lettuce Leaf’, ‘Comune’ and also ‘Comune’ cultivars were used for basil, mint, and sage, respectively. These cultivars were selected as representatives of the basil, mint, and sage cultivated in the south Mediterranean region including Italy, Spain, and Greece. 

**Figure 8:**
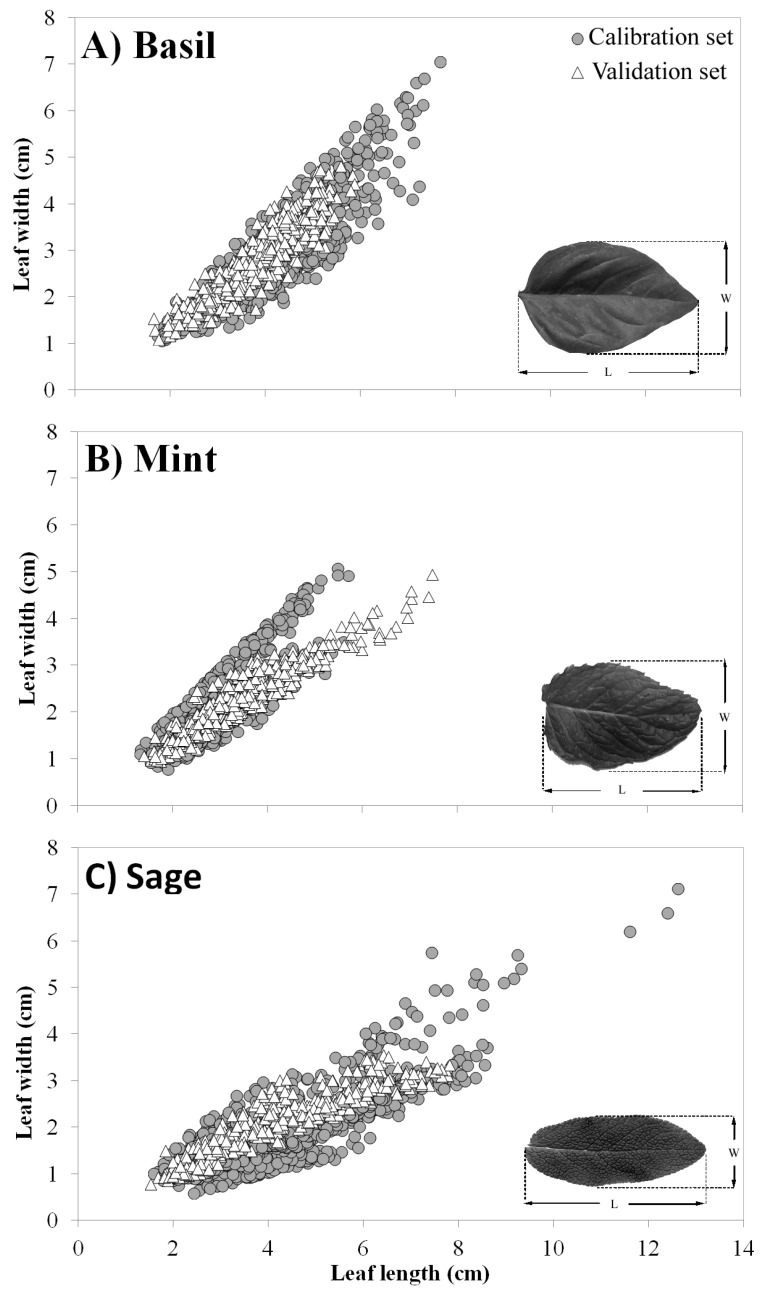
Leaf length vs. leaf width of the calibration (grey circle) and validation (white triangle) sets of the three aromatic species: (**A**) basil, (**B**) mint, and (**C**) sage selected in this work. The plots are also reported the leaf shapes of the three aromatic species showing the position of leaf length (L) and width (W).

A total of 206 to 235 healthy leaves were collected for each of the 14 cultivars used for model calibration, whereas 400 to 500 healthy leaves were collected for each of the 3 cultivars used for model validation. Leaves with a minimum blade width of 0.5 cm were randomly collected in spring from different levels of the canopy in order to capture the natural variability in the leaf shape of each cultivar. Collected leaves were rapidly transported to the laboratory where the parameters L, W, and LA of the leaf blades were individually measured (Figure 8). LA was measured with an LA-meter (LI-3100; LICOR, Lincoln, NE, USA) calibrated to 0.01 cm^2^.

### 4.2. Statistical Analysis

All the measurements were carried out using R-STAT (a free software environment for statistical computing and graphics; version n. 3.5.2, release codenamed “Eggshell Igloo”; © 2018 of The R Foundation for Statistical Computing) and packages available in the CRAN (comprehensive R archive network) repository [51].

#### 4.2.1. Principal Component Analysis

To understand how different phenotypic traits such as leaf L and W influenced the single LA of different cultivars within each species, the Pearson correlation between the three parameters was estimated using the cor() function of the ‘Stats’ package [51]. Moreover, a principal component analysis (PCA), based on the singular value decomposition (SVD) [52], was carried out following the indications of Manly and Alberto [53], using the prcomp() function, also available in the ‘Stats’ package [51].

#### 4.2.2. Model Calibration

Five different allometric models (3 linear and 2 quadratic) were chosen based on leaf phenotypic traits [26] of the 3 selected species, and were used to estimate the single LA based on fast measurements of 2 proxy variables as follows:

LA = *a* + *b* × L (1)

LA = *a* + *b* × W (2)

LA = *a* + *b* × (L × W) (3)

LA = *a* + *b* × L^2^ (4)

LA = *a* + *b* × W^2^ (5)

where *a* and *b* are the coefficients of the linear and quadratic models, LA is the single leaf area (cm^2^), L is the leaf length (cm), and W is the leaf width (cm).

Regarding basil, LA estimation was carried out by aggregating data (calibration set, *n* = 1094; Table 6) measured on 5 cultivars, i.e., ‘Aroma’, ‘Cinnamon’, ‘Mammoth’, ‘Purple Petra’ and ‘Super Sweet Chen’. In the case of mint, the pooled data (calibration set, *n* = 901; Table 6) were collected on 4 cultivars, namely ‘Glaciale’, ‘Moroccan’, ‘Piperita’ and ‘Suaveolens’. The pooled calibration dataset (*n* = 1103; Table 6) for sage was based on data collected on 5 cultivars, i.e., ‘Fariancea’, ‘Jcterina’, ‘Maxima’, ‘Purpurea’ and ‘Tricolor’.

The linear and quadratic models were fitted using the lm() function of the ‘Stats’ package [51]. The following criteria were used to evaluate the performance of the 5 allometric models: R-square (R²), root mean square error (RMSE in cm^2^) and the Bayesian information criterion (BIC) [54]. The BIC() function, available in the ‘Stats’ [51] package was used to calculate the BIC criterion. Finally, the best ranking model, both for the one-regressor and two-regressors models, was chosen as that having the highest R^2^ and the lowest RMSE and BIC. Therefore, the coefficients of the selected models were optimized using a non-parametric bootstrap function available on the ‘Boot’ package [55]. The coefficients of the bootstrap model together with the associated standard, median, and percentage confidence intervals were obtained iteratively in 1000 selected bootstrap samples with substitution of observations from the original data set.

#### 4.2.3. Model Validation

The selected bootstrapped models (with one or double predictors) were validated by comparing the predicted single leaf area (PLA) values estimated using L and/or W values, obtained during the validation experiment with the observed single LA (OLA) values of the following cultivars:

1. Cultivar ‘Lettuce Leaf’ (*n* = 487) for basil;
2. Cultivar ‘Comune’ (*n* = 441) for mint;
3. Cultivar ‘Comune’ (*n* = 418) for sage.

The goodness of estimation of the selected models was evaluated by analyzing the R² and the RMSE (cm^2^) of the observed leaf area (OLA) compared to the predicted leaf area (PLA) for each cultivar used to validate each model.

## 5. Conclusions

Much research has been conducted on several fruit crops and MAPs to characterize leaf traits. In addition, numerous models based generally on two important proxy parameters, such as leaf L and/or W have been proposed, tested, and validated. In this study, single LA of basil, mint and sage, was predicted using five allometric models (linear or quadratic) based both on leaf L or W or on the product of these two variables. Our results confirmed the capability of two-regressors models, especially when intra-specific variability is considered, to estimate single LA for the abovementioned species. Indeed, this study suggested that by using a two-regressors model based on the product of leaf W and L, predictions can be quite accurate without the need to calibrate model coefficients for genotype. Nevertheless, calibration might be required if the selected cultivars are growing under biotic and/or abiotic stress conditions.

On the other hand, even if a good fitting can be found using the one-regressor model based on squared leaf W (model no. 5), predictions (especially in the case of mint and sage) might be biased.

Eventually, estimates of single LA of basil, mint, and sage, obtained with different levels of accuracy using one or more parameters and the validated coefficients can be a valid resource for plant physiologists, horticulturists, and growers to better estimate the single leaf area and the seasonal variation of LAI of their crops, to parameterize models (e.g., to estimate evapotranspiration) or to extract general information regarding the vigor and the growth stage of their crops. Since two-regressors models allowed very accurate estimations of leaf blade size of basil, sage, and mint, they can be useful also to support plant physiology studies and to develop more complex process-based plant growth models that can be used to build support decision systems to help growers in managing fertilization and irrigation.

## Figures and Tables

**Table 1:** Characteristics of phenotypic traits (L, W, and L:W) of aromatic species (basil, mint, and sage) and cultivars used in this study.

Species and Cultivar		Leaf Length (L; cm)				Leaf Width (W; cm)				Leaf Shape Ratio (L:W)			
	No. of Leaves Sampled	Min	Median	Mean	Max	Min	Median	Mean	Max	Min	Median	Mean	Max
Basil (*Ocimum basilicum* L.)													
Aroma	214	1.92	3.90	3.85	5.77	1.26	2.49	2.47	3.95	1.22	1.56	1.57	2.06
Cinnamon	222	1.81	3.76	3.80	7.23	1.06	2.35	2.38	4.37	1.17	1.60	1.60	2.16
Lettuce Leaf	487	1.65	3.90	3.85	5.87	1.09	2.82	2.83	4.81	1.03	1.24	1.27	1.76
Mammoth	217	1.95	5.07	4.98	7.66	1.41	4.08	3.98	7.05	1.24	1.53	1.53	2.16
Purple Petra	235	1.73	3.24	3.18	4.37	1.09	2.15	2.09	3.18	1.22	1.64	1.66	2.41
Super Sweet Chen	206	1.87	4.10	4.10	5.93	1.12	2.57	2.52	4.14	1.04	1.37	1.39	2.17
Mint (*Mentha* spp.)													
Comune	441	1.40	3.47	3.57	7.47	0.96	2.35	2.36	4.95	1.00	1.09	1.11	1.37
Glaciale	230	1.32	2.95	3.06	5.70	1.11	2.70	2.79	5.08	1.10	1.44	1.44	1.83
Moroccan	229	1.59	3.21	3.26	5.07	1.19	2.32	2.28	3.50	1.12	1.57	1.58	2.25
Piperita	222	1.76	2.93	3.06	5.64	1.02	1.93	1.96	3.51	1.26	1.79	1.76	2.42
Suaveolens	220	1.53	2.32	2.37	3.37	0.78	1.36	1.35	1.84	1.02	1.53	1.52	2.04
Sage (*Salvia* spp.)													
Fariancea	231	2.10	4.29	4.58	7.81	0.58	1.26	1.51	3.21	2.05	3.41	3.23	4.16
Jcterina	216	1.98	3.84	4.00	7.97	0.85	1.54	1.62	3.06	1.68	2.48	2.47	3.22
Maxima	223	1.59	3.86	4.40	12.61	0.86	2.38	2.62	7.11	1.24	1.67	1.68	2.30
Comune	418	1.52	4.05	4.22	7.80	0.78	2.13	2.11	3.55	1.96	2.43	2.43	3.08
Purpurea	222	1.97	4.93	5.05	8.60	0.89	1.96	2.08	3.78	1.63	2.26	2.26	2.90
Tricolor	211	1.84	3.54	3.93	6.53	0.87	1.61	1.73	3.01	1.22	2.03	2.00	2.54

**Table 2:** Pearson correlation values between dependent (LA) and independent variables (L, W) used in the calibration models for aromatic species (basil, mint, and sage) and cultivars. LA = single leaf area (cm^2^); L = leaf length (cm); W = leaf width (cm).

Cultivar	LA vs. W	LA vs. L
Basil (*Ocimum basilicum* L.)		
Aroma	0.974	0.955
Cinnamon	0.956	0.963
Mammoth	0.975	0.947
Purple Petra	0.977	0.965
Super Sweet Chen	0.973	0.936
Mean	0.971	0.953
Mint (*Mentha* spp.)		
Glaciale	0.979	0.985
Moroccan	0.959	0.975
Piperita	0.959	0.957
Suaveolens	0.877	0.930
Mean	0.944	0.962
Sage (*Salvia* spp.)		
Fariancea	0.987	0.955
Jcterina	0.973	0.973
Maxima	0.958	0.961
Purpurea	0.983	0.982
Tricolor	0.981	0.979
Mean	0.976	0.970

**Table 3:** Statistical outputs from the principal component analysis based on different variables (LA, L, W) for three aromatic species. LA = single leaf area (cm^2^); L = leaf length (cm); W = leaf width (cm).

Parameter	PC1	PC2	PC3	PC1	PC2	PC3	PC1	PC2	PC3
	Basil			Mint			Sage		
Eigenvalue	2.864	0.115	0.021	2.819	0.164	0.017	2.778	0.175	0.047
Standard deviation	1.692	0.339	0.145	1.679	0.405	0.132	1.667	0.418	0.216
Proportion of Variance	0.955	0.038	0.007	0.940	0.055	0.006	0.926	0.058	0.016
Cumulative Proportion of Variance	0.955	0.993	1.000	0.940	0.994	1.000	0.926	0.984	1.000
	Loading								
LA	−0.585	0.286	−0.759	0.592	−0.099	−0.800	0.589	−0.199	−0.783
L	−0.568	−0.813	0.132	0.566	0.757	0.326	0.565	0.794	0.223
W	−0.579	0.508	0.637	0.574	−0.646	0.504	0.577	−0.574	0.580

**Table 4:** Fitted coefficient (*b*) and constant (*a*) values of the models used to estimate the single leaf area (LA) of the three aromatic species from length (L) and width (W) measurements; coefficient of determination (R^2^), root mean square error (RMSE in cm^2^) and Bayesian Information Criterion (BIC) of the various models are also given. The standard errors (SE) and *p*-value in parenthesis. L and W were in cm. All data were derived from the calibration experiment. The one- and two-regressors models with the best ranking are reported in a grey cell, whereas the best values for each selection criterion are reported in bold.

Model No.	Form of the Model Tested	Constant and Fitted Coefficient		*R* ^2^	RMSE	BIC
		*a* (SE/*p*-Value)	*b* (SE/*p*-Value)			
Basil (*Ocimum basilicum* L.)						
1	LA = *a* + *b* × L	−10.20 (0.24/ ***)	4.62 (0.06/ ***)	0.850	2.10	4751.4
2	LA = *a* + *b* × W	−6.47 (0.10/ ***)	5.45 (0.04/ ***)	0.954	1.17	3459.0
**3**	LA = *a* + *b* × (L × W)	−0.12 (0.02/ ***)	0.71 (0.01/ ***)	**0.995**	**0.40**	**1122.6**
4	LA = *a* + *b* × L^2^	−1.28 (0.10/ ***)	0.56 (0.01/ ***)	0.909	1.64	4201.1
**5**	LA = *a* + *b* × W^2^	1.61 (0.05/ ***)	0.80 (0.01/ ***)	**0.956**	**1.14**	**3413.3**
Mint (*Mentha* spp.)						
1	LA = *a* + *b* × L	−5.92 (0.15/ ***)	3.71 (0.05/ ***)	0.861	1.22	2928.7
2	LA = *a* + *b* × W	−3.39 (0.09/ ***)	3.99 (0.04/ ***)	0.922	0.91	2410.1
**3**	LA = *a* + *b* × (L × W)	0.03 (0.01/ **)	0.74 (0.00/ ***)	**0.997**	**0.17**	**−620.9**
4	LA = *a* + *b* × L^2^	−0.36 (0.07/ ***)	0.58 (0.01/ ***)	0.888	1.09	2733.3
**5**	LA = *a* + *b* × W^2^	1.06 (0.05/ ***)	0.78 (0.01/ ***)	**0.925**	**0.89**	**2374.6**
Sage (*Salvia* spp.)						
1	LA = *a* + *b* × L	−8.00 (0.24/ ***)	3.37 (0.05/ ***)	0.791	2.69	5333.5
2	LA = *a* + *b* × W	−5.94 (0.15/ ***)	6.67 (0.07/ ***)	0.891	1.94	4610.9
**3**	LA = *a* + *b* × (L × W)	−0.04 (0.02/ns)	0.72 (0.00/ ***)	**0.994**	**0.45**	**1373.2**
4	LA = *a* + *b* × L^2^	−0.62 (0.10/ ***)	0.34 (0.00/ ***)	0.888	1.96	4639.2
**5**	LA = *a* + *b* × W^2^	1.30 (0.06/ ***)	1.27 (0.01/ ***)	**0.937**	**1.48**	**4011.7**

Note: *** = *p* < 0.001; ** = *p* < 0.01; ns = not significant.

**Table 5:** Main outputs for non-parametric bootstrap analysis (replications: 1000) of models no. 3 and no. 5 fitted with data of single leaf area (LA in cm^2^), leaf length (L) and width (W) measurements collected from different cultivars of three aromatic species; SE is the bootstrapped standard error; root mean square error (RMSE) in cm^2^.

Model No.	Dependent Variable	Number of Regressors	Parameter ^†^	Original		Boot				Percent Confidence Interval	
				Value	(*p*-Value)	Value	Bias	SE	Median	2.5%	97.5%
Basil (*Ocimum basilicum* L.)											
3	LA	2	R^2^	0.995	-	0.995	0.000	0.000	-	-	-
			RMSE	0.401	-	0.370	−0.030	0.114	-	-	-
			(intercept)	−0.125	(***)	−0.125	0.000	0.022	−0.127	−0.184	−0.068
			L × W	0.713	(***)	0.713	0.000	0.002	0.713	0.707	0.719
5	LA	1	R^2^	0.956	-	0.956	0.000	0.003	-	-	-
			RMSE	1.142	-	1.104	−0.038	0.265	-	-	-
			(intercept)	1.612	(***)	1.603	−0.008	0.055	1.614	1.477	1.735
			W^2^	0.802	(***)	0.803	0.001	0.005	0.802	0.786	0.822
Mint (*Mentha* spp.)											
3	LA	2	R^2^	0.997	-	0.997	0.000	0.000	-	-	-
			RMSE	0.170	-	0.156	−0.013	0.044	-	-	-
			(intercept)	0.030	(**)	0.030	0.000	0.010	0.029	0.006	0.053
			L × W	0.739	(***)	0.739	0.000	0.001	0.739	0.735	0.743
5	LA	1	R^2^	0.925	-	0.925	0.000	0.006	-	-	-
			RMSE	0.895	-	0.840	−0.055	0.231	-	-	-
			(intercept)	1.060	(***)	1.058	−0.002	0.048	1.061	0.978	1.136
			W^2^	0.781	(***)	0.782	0.000	0.007	0.781	0.763	0.803
Sage (*Salvia* spp.)											
3	LA	2	R^2^	0.994	-	0.994	0.000	0.001	-	-	-
			RMSE	0.447	-	0.414	−0.033	0.142	-	-	-
			(intercept)	−0.036	(ns)	−0.035	0.002	0.021	−0.036	−0.078	0.009
			L × W	0.723	(***)	0.723	0.000	0.002	0.723	0.717	0.729
5	LA	1	R^2^	0.937	-	0.936	−0.001	0.010	-	-	-
			RMSE	1.478	-	1.352	−0.126	0.435	-	-	-
			(intercept)	1.304	(***)	1.308	0.004	0.062	1.293	1.147	1.487
			W^2^	1.270	(***)	1.269	−0.001	0.010	1.273	1.219	1.317

Note: *** = *p* < 0.001; ** = *p* < 0.01; ns = not significant; ^†^ L and W were in cm.

**Table 6:** Minimum, maximum, and mean values of phenotypic traits (L, W, L × W, L:W) and single-leaf area (LA) of aromatic species (basil, mint, and sage) and cultivars used in this study.

Group	No. of Cultivars	No. of Leaves Sampled	L (cm)			W (cm)			L × W (cm^2^)			L:W			LA (cm^2^)		
			Min	Max	Mean	Min	Max	Mean	Min	Max	Mean	Min	Max	Mean	Min	Max	Mean
Basil (*Ocimum basilicum* L.)																	
Calibration set	5	1094	1.73	7.66	3.97	1.06	7.05	2.68	1.92	54.00	11.57	1.03	2.41	1.53	1.40	37.39	8.12
Validation set	1	487	1.65	5.87	3.85	1.09	4.81	2.83	1.90	26.92	11.46	1.04	2.17	1.39	1.30	18.90	8.06
Mint (*Mentha* spp.)																	
Calibration set	4	901	1.32	5.70	2.94	0.78	5.08	2.10	1.42	28.05	6.73	1.00	2.42	1.47	1.07	20.45	5.00
Validation set	1	441	1.40	7.47	3.57	0.96	4.95	2.36	1.50	37.01	9.16	1.02	2.04	1.52	1.08	27.27	6.81
Sage (*Salvia* spp.)																	
Calibration set	5	1103	1.59	12.61	4.40	0.58	7.11	1.91	1.41	89.64	9.49	1.24	4.16	2.42	1.01	64.55	6.83
Validation set	1	418	1.52	7.80	4.22	0.78	3.55	2.11	1.19	26.14	9.83	1.22	2.54	2.00	0.94	19.70	7.39

## Supplementary Materials

The following are available online at https://www.mdpi.com/2223-7747/9/1/13/s1, Figure S1: Analysis of dispersion pattern of residuals for models no. 5 (A) and no. 3 (B). Figure S2: Analysis of dispersion pattern of residuals for models no. 5 (A) and no. 3 (B). Figure S3: Analysis of dispersion pattern of residuals for models no. 5 (A) and no. 3 (B) are shown in the insets.
