# Supplementary material for: Analysis of Cultivar-Specific Variability in Size-Related Leaf Traits and Modeling of Single Leaf Area in Three Medicinal and Aromatic Plants: Ocimum basilicum L., Mentha Spp., and Salvia Spp
Source: Plants (Basel). 2019 Dec 20;9(1):13. doi: 10.3390/plants9010013 (PMC7020212; doi:10.3390/plants9010013)
Supplement: Supplementary file 1 [file plants-09-00013-s001.pdf]

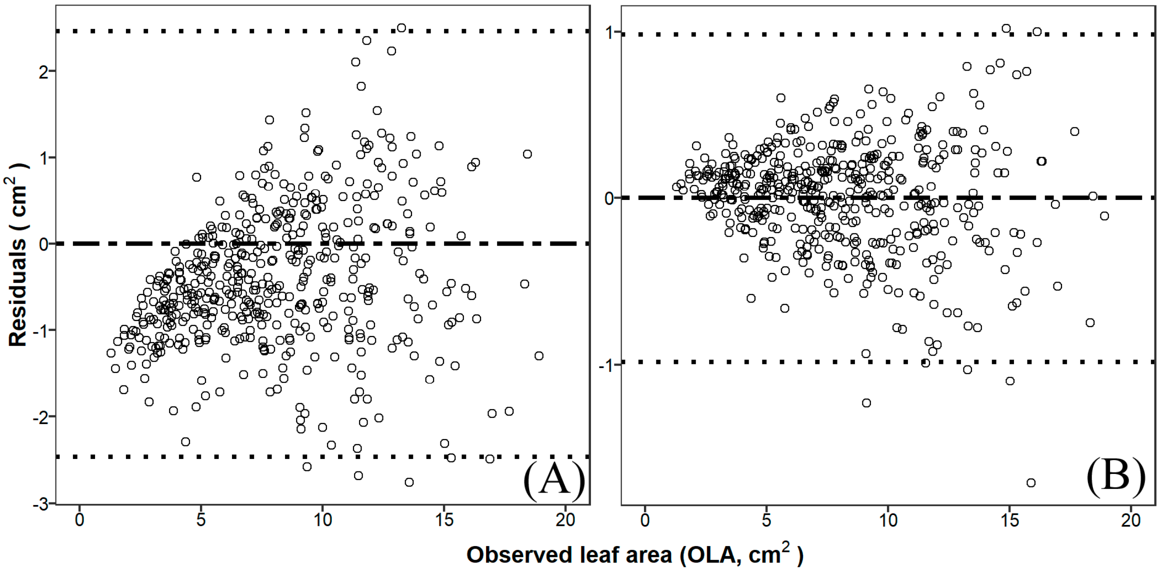

Figure S1. Analysis of the dispersion pattern of residuals for models no. 5 (**A**) and no. 3 (**B**). Residuals = the difference between predicted leaf areas (PLA) estimated by model no. 3 or no. 5 (with coefficients obtained from pooled data from 5 basil cultivars, see Table 1 for more details) vs. the observed leaf area of basil 'Lettuce Leaf' cultivar. In each plot, the horizontal dash-dotted line represents the zero residual line, whereas the two horizontal dashed lines are the limits of agreement, calculated as d ± 3 SD (where d is the mean of the differences, and SD is the standard deviation of the differences).

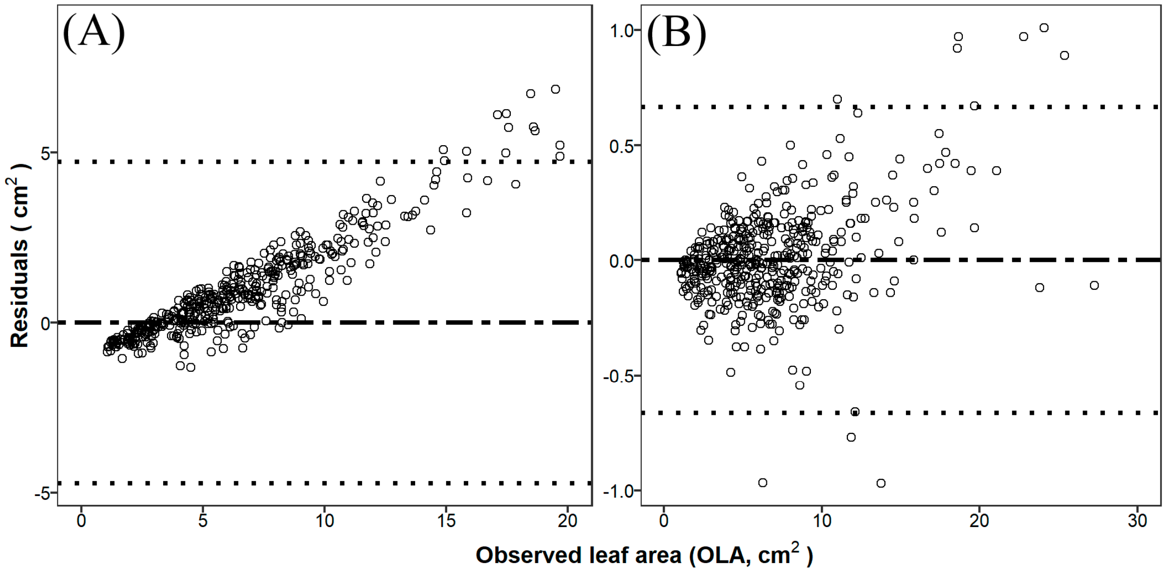

Figure S2. Analysis of dispersion pattern of residuals for models no. 5 (**A**) and no. 3 (**B**). Residuals = the difference between predicted leaf areas (PLA) estimated by model no. 3 or no. 5 (with coefficients obtained from pooled data from 4 mint cultivars, see Table 1 for more details) vs. the observed leaf area of mint 'Comune' cultivar. In each plot, the horizontal dash-dotted solid line represents the zero residual line, whereas the two horizontal dashed lines are the limits of agreement, calculated as d ± 3 SD; (where d is the mean of the differences, and SD is the standard deviation of the differences).

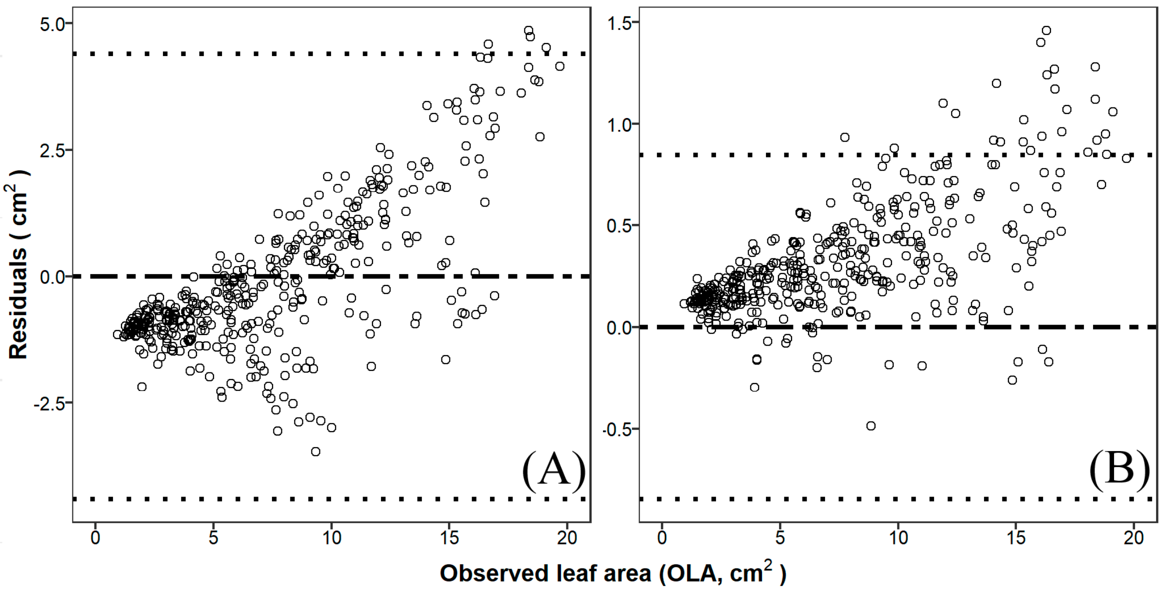

Figure S3. Analysis of dispersion pattern of residuals for models no. 5 (**A**) and no. 3 (**B**) are shown in the insets. Residuals = the difference between predicted leaf areas (PLA) estimated by model no. 3 or no. 5 (with coefficients obtained from pooled data from 5 sage cultivars, see Table 1 for more details) vs. the observed leaf area of sage 'Comune' cultivar. In each plot, the horizontal dash-dotted solid line represents the zero residual line, whereas the two horizontal dashed lines are the limits of agreement, calculated as d ± 3 SD; (where d is the mean of the differences, and SD is the standard deviation of the differences).
